# A novel setup approach for helical tomotherapy in head and neck cancer: A case report

**DOI:** 10.3892/ol.2013.1558

**Published:** 2013-09-02

**Authors:** MARCIANA NONA DUMA, HANS GEINITZ, SEVERIN KAMPFER, MARCO R. KESTING

**Affiliations:** 1 Department of Radiation Oncology, Technical University of Munich, Klinikum rechts der Isar, Munich, Germany; 2 Department of Radiation Oncology, Krankenhaus der Barmherzigen Schwestern Linz, Linz, Austria; 3 Department of Oral and Maxillofacial Surgery, Technical University of Munich, Klinikum rechts der Isar, Munich, Germany

**Keywords:** head and neck cancers, helical tomotherapy, recurrence, image guided radiotherapy, setup

## Abstract

Head and neck cancers that are associated with high-risk factors have a poor prognosis. In such patients, post-operative radiochemotherapy is mandatory. The present study describes the case of a 71-year-old high-risk head and neck cancer patient who was not able to tolerate the supine position for the radiotherapy setup. A lateral immobilization with a head mask and a vacuum cushion was performed. The patient underwent daily computed tomography (CT)-guided radiation therapy [image-guided radiation therapy (IGRT)]. At nine months post-radiotherapy, the patient had no xerostomia and no swallowing dysfunction. However, the patient suffered a local recurrence and succumbed due to bleeding of the tumor a number of weeks after the recurrence. A recalculation of the actual delivered dose, taking the daily delivered dose into account, was performed. The recurrence occurred within the high-dose region. In selected cases of patients with head and neck cancers who are unable to tolerate the supine position, lateral positioning and high precision treatment is possible using daily IGRT.

## Introduction

The benefit of a multidisciplinary approach in head and neck cancer patients was demonstrated by a phase III trial from the UK that was published in the late nineties. In this trial, 350 T2-T4 N0-N2 oral cavity or oropharynx cancer patients were randomized to either undergo surgery and post-operative conformal radiotherapy or irradiation alone. The trial was closed early, as a significant difference in favor of the combined treatment arm was noted at 23 months for overall survival, cause-specific survival and local control (1). In head and neck cancers that are associated with high-risk factors, including lymph node involvement with extracapsular extension (ECE), post-operative radiochemotherapy is mandatory.

However, post-operative 3D conformal radiotherapy (3D-CRT) has certain limitations, including xerostomia, mandible necrosis and trismus. These toxicities may be reduced by using intensity-modulated radiotherapy (IMRT) (2–4).

Furthermore, repeated computed tomography (CT) scans may be performed during the course of radiotherapy directly in the treatment room in order to optimize the position of the patient prior to administering IMRT [image-guided radiation therapy (IGRT)]. Therefore, potential losses in the local control due to missing the target may be avoided (5,6).

The present case is an example of how IGRT allows a safe dose delivery of highly conformal treatment plans for rare patient setups in head and neck radiotherapy. Written informed consent was obtained from the patient’s family.

## Case report

### Presentation

A 71-year-old male, with a body weight of 50 kg and a height of 1.62 m, presented with a visible superficial ulcer with poorly-defined margins on the right side of the tongue. The patient complained of oral pain and dysphagia that had lasted for several months. The patient had a history of drinking 1 beer/day and a smoking history of 30 pack years. The past medical history included heavy chronic obstructive pulmonary disease (COPD), pulmonary tuberculosis, arterial hypertension, peripheral arterial occlusive disease with bilateral stenosis of the internal carotid arteries and liver cirrhosis. The biopsy revealed a poorly-differentiated squamous cell carcinoma of the floor of the mouth.

### Treatment

The surgery involved a tracheotomy to assure the airways remained open and a dissection of levels I–IV on the right side and I–III on the left side of the neck. A radical surgical tumor excision and reconstruction, with a microvascular anastomosed free flap from the anterolateral thigh (ALT), was then performed. Due to continued heavy tracheal secretion as a result of severe COPD, the tracheal cannula was not removed.

**Figure 1:**
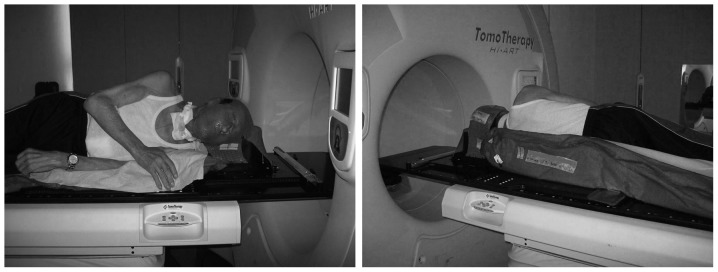
Lateral positioning for radiotherapy. A head mask and a vacuum cushion were fit to the patient. The vacuum cushion was fitted in order to support the back of the patient.

The definitive tumor-node-metastasis (TNM) status was pT2 (2.5 cm) pN2b (3/39 with extracapsular extension) G3 Pn1 L0 V0 R0 (≥0.2 cm to the floor of the mouth, ≥1 cm to all the other sites). The multidisciplinary head and neck tumor board recommended adjuvant therapy. Prior to adjuvant therapy, the patient underwent a percutaneous endoscopic gastrostomy (PEG). Every time the patient was placed in the supine position during the radiotherapy setup, the patient experienced severe coughing attacks. No coughing was observed while sitting or lying on the side in a lateral position. Several changes of the tracheal cannula did not improve the coughing. A lateral position was decided upon (Fig. 1) utilizing a head mask and a vacuum cushion (BodyFIX; Medical Intelligence, Schwabmünchen, Germany).

Due to the age of the patient, comorbidities and insufficient renal function (clearance 47 ml/min), no platinum-based chemotherapy was administered.

### Results

**Figure 2:**
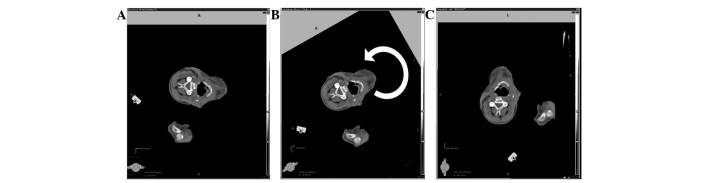
Rotation of axial slices for radiotherapy planning target volume contouring. (A) CT image in lateral position as used for radiation treatment. (B) Rotation of CT image. (C) Rotated CT image from A.

In order to contour the planning target volume (PTV), software was required that allowed the rotation of the CT images at 90 degrees to the normal view that radiation oncologists are used to while contouring (Fig. 2). The contouring was performed using Eclipse (Varian Medical Systems, Palo Alto, CA, USA). The tumor clinical target volume (CTV) was defined as the gross tumor volume (GTV) delineated on a pre-operative CT, including the visible post-operative changes on the post-operative CT plus a safety margin of 10 mm. The elective nodal-CTV was defined according to the literature (7,8).

**Figure 3:**
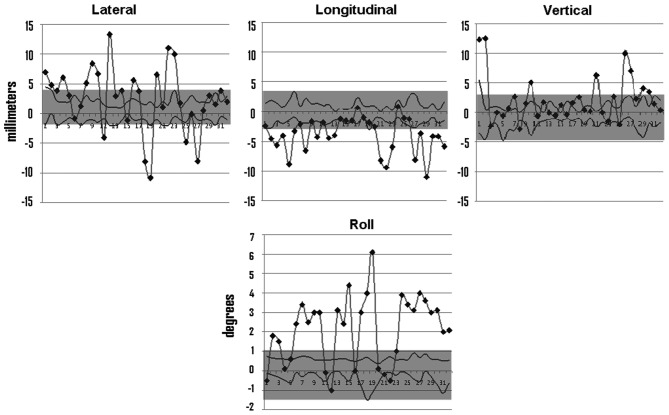
Daily setup errors of the present case corrected by image guidance (IG) in the lateral, longitudinal and vertical directions, as well as the roll rotation. Furthermore, the graph depicts the ranges of the setup errors of 20 head and neck cancer patients that were treated using a supine position with tomotherapy in Klinikum rechts der Isar (Munich, Germany).

In-ward radiotherapy was performed using a TomoTherapy machine (Accuray Incorporated, Sunnyvale, CA, USA). Daily image guidance (IG) was performed using an incorporated mega-voltage CT (MVCT). The overall treatment time was 6.5 weeks. The daily setup errors in the translational directions and the rotational direction (roll) are presented in Fig. 3.

The patient tolerated the treatment well. At the end of the radiotherapy, the patient presented with Common Terminology Criteria for Adverse Events (CTCAE) grade 3 mucositis and CTCAE grade 1 dermatitis. A combined PEG-oral food intake was possible during the entire treatment time.

### Follow-up

The patient underwent regular follow-up sessions every three months. Seven months after the surgery, the patient had experienced no more problems with deglutition and the tracheotomy was closed. At nine months after radiotherapy, the patient had no xerostomia, no swallowing dysfunction, localized submental edema, mild voice alteration and moderate skin induration. However, the CT revealed a tumor recurrence at the floor of the mouth, which was histologically proven.

**Figure 4:**
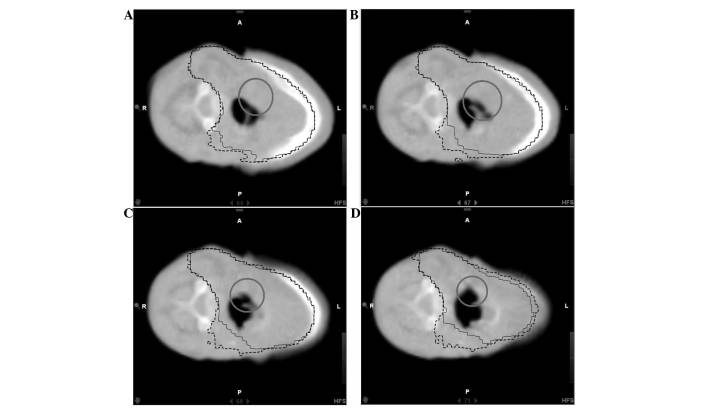
Planned and actual delivered dose. The solid line corresponds to the planned 95% isodose (60.8 Gy) and the dashed line to the actual delivered 95% isodose. The circle corresponds to the tumor recurrence site. Depicted are CT slices at different levels: (A) slice number 65; (B) slice number 67; (C) CT slice number 69; (D) CT slice number 71.

An evaluation of the planned dose was insufficient in this case. An assessment of the impact of setup and soft tissue shrinkage on the actual daily delivered dose was necessary. Therefore, retrospective recalculations of the doses were performed on each of the 32 daily MVCTs. All the daily doses were summed up (9). Fig. 4 depicts the actual delivered 95% isodose on the last MVCT. The recurrence was localized in field, within the 95% isodose.

The multidisciplinary head and neck board recommended palliative chemotherapy, however the patient succumbed due to tumor bleeding prior to the start of chemotherapy.

## Discussion

The present case is an example of how novel radiotherapy techniques may be used to enhance head and neck oncological treatment.

Due to severe coughing attacks that were caused by the supine position, the patient in the present study was not able to be treated in the standard supine position. Several options were discussed, including not performing radiotherapy treatment at all or choosing a prone or lateral positioning for radiotherapy. Due to the high-risk tumor constellation, a local treatment was considered necessary. However, few immobilization devices are available that allow a mask fixation in an alternative position to the supine one. There is a mask system available for craniospinal irradiation that permits a prone position with a head mask (10), however, the use of a prone position is not possible with a patient with a PEG, as they are consequently not able to lie on their abdomen.

Therefore, an individualized, lateral position was chosen. The lateral position, though tolerated well by the patient, raised several problems with regard to the reproducibility of the setup. The lateral position is more unstable than the supine position (11). Thus, there was a risk of inadvertently delivering a high dose to healthy tissue. Therefore, the options were either to deliver a lower dose to the PTV by 3D-CRT, with a lower probability of achieving local control, or to deliver the standard dose with an increased risk of toxicity. IMRT is known to reduce toxicity (3,4) without compromising the dose to the target volumes. Taking the high-risk situation into account and the importance of adjuvant treatment, IMRT (helical tomotherapy) was performed with delineation and dose prescription according to the available guidelines for standard supine head and neck treatment (7,8). Daily CT-guided IGRT was performed in order to ensure the reproducibility of setup.

The setup errors for this patient (Fig. 3) were higher than the reported setup errors for the supine position in head and neck radiotherapy, which are normally within 2–4 mm (12,13). Thus, the decision to treat such a patient with IMRT without daily IGRT would have been risky.

The combination of IMRT with daily IGRT translated into low toxicities at the follow-up appointments. No xerostomia or swallowing dysfunction and only a mild submental edema were observed.

However, the tumor recurred. The question was raised as to whether the setup uncertainties, although compensated by daily CT-IGRT, in combination with the steep dose gradients of helical tomotherapy may have caused an underdosage of the planning target regions. A simple correlation of the recurrence site with the dose on the planning CT would have been incomplete for several reasons. Firstly, the patient may have had a slight alternative position during radiotherapy compared with the planning CT. Secondly, head and neck patients often undergo soft tissue changes during radiotherapy (5).

Generally, if it may be assumed that patients are rigid bodies, setup uncertainties may be perfectly compensated for by daily IGRT. However, deformations of organs occur during fractionated radiotherapy, including a slightly varied curvature of the spinal cord, an alternative position of the chin and shoulders and/or shrinkage of the soft tissues. A perfect alignment, in which the organs lie exactly in the same position as during the planning CT and thus the delivered dose is exactly the same as the calculated dose on the planning CT, is not feasible.

In order to determine the impact of interfractional uncertainties and of soft tissue deformations on the delivered dose, the actual delivered dose was recalculated on every daily MVCT and all the daily delivered doses were summed up. The summed dose was not completely accurate, as non-rigid registration was not used. However, the dose represented an approximation of the actual delivered dose. The recurrence was localized in-field, within the 95% actual delivered isodose (Fig. 4). This is consistent with other studies on the recurrence sites of head and neck tumors in association with the isodoses on the planning CT (14). Thus, lateral positioning with daily-corrected setup errors did not jeopardize the accurate delivery of the dose to the target volume.

In selected cases of patients with head and neck cancers who are unable to tolerate the supine position, lateral positioning and high precision treatment is possible using daily IGRT.
